# Noninvasive quantitation of rat cerebral blood flow using ^99m^Tc-HMPAO—assessment of input function with dynamic chest planar imaging

**DOI:** 10.1186/s13550-018-0375-7

**Published:** 2018-03-09

**Authors:** Chie Suzuki, Mutsumi Kosugi, Yasuhiro Magata

**Affiliations:** Preeminent Medical Photonics Education and Research Center, Hamamatsu University School of Medicine, 1-20-1 Handayama, Higashi-ku, Hamamatsu, Shizuoka 431-3192 Japan

**Keywords:** Cerebral blood flow, Image-derived input function, Planar imaging, Rat, ^99m^Tc-HMPAO

## Abstract

**Background:**

Cerebral blood flow (CBF) quantitation using technetium-99m hexamethylpropyleneamine oxime (^99m^Tc-HMPAO) generally requires assessment of input function by arterial blood sampling, which would be invasive for small animals. We therefore performed chest dynamic planar imaging, instead of arterial blood sampling, to estimate the input function and establish noninvasive quantitation method of rat CBF using the image-derived input function.

**Results:**

Integrated radioactivity concentration in the heart-blood pool on planar images (AUC_Blood_-planar) was identical to that in arterial blood samples (AUC_Blood_-sampling). Radioactivity concentration in the brain determined by SPECT imaging (C_Brain_-SPECT) was identical to that using brain sampling (C_Brain_-sampling). Noninvasively calculated CBF obtained by dividing C_Brain_-SPECT by AUC_Blood_-planar was well correlated with conventionally estimated CBF obtained by dividing C_Brain_-sampling by AUC_Blood_-sampling.

**Conclusion:**

Rat CBF could be noninvasively quantitated using ^99m^Tc-HMPAO chest dynamic planar imaging and head SPECT imaging without arterial blood sampling.

## Background

Cerebral blood flow (CBF) is associated with brain function in acute and chronic brain disorders, such as cerebrovascular disease, dementia, and epilepsy [1]. CBF levels also influence drugs’ therapeutic efficacy because most drug delivery to the brain is dependent on CBF. For studies using small-animal models of these brain disorders, CBF assessment meaningfully enhances the understanding of pathology in model animals and facilitates evaluation of drug efficacy.

Tc-99m hexamethylpropyleneamine oxime (^99m^Tc-exametazime, ^99m^Tc-HMAO) is a single-photon emission computed tomography (SPECT) radiotracer for CBF assessment [2]. Our previous study reported that rat CBF could be quantified using ^99m^Tc-HMPAO [3]. Quantitative assessment of CBF using ^99m^Tc-HMPAO requires input function, which is generally assessed by arterial blood sampling. Arterial cannulation would be invasive for small animals and difficult to repeat in the same animal over the long term [4]. In addition, abundant and frequent blood sampling can affect the physiological condition, including blood pressure, blood cell count, and plasma hormone levels [5, 6]. Although blood sampling systems using microfluidic technologies have been developed to reduce the amount of blood sampling from small animals [4, 7], they cannot entirely obviate the need for arterial cannulation and blood sampling. Therefore, evaluation of the input function without blood sampling would be desirable for CBF quantification in small animals.

Input function assessed by dynamic imaging of a large blood pool is used for quantitative analysis of some SPECT and positron emission tomography (PET) radiotracers in humans [8–10] and small rodents [11]. In the present study, we performed dynamic planar imaging of the heart after intravenous administration of ^99m^Tc-HMPAO to assess image-derived input function. Rat CBF was quantified using the input function obtained from dynamic planar images and compared with the CBF conventionally quantified using arterial blood sampling.

## Methods

Sodium [^99m^Tc]pertechnetate was eluted from a ^99^Mo/^99m^Tc generator (Ultra Techne Kow, Fujifilm RI Pharma Co., Ltd., Tokyo, Japan), which was previously eluted within 24 h. The kit formulation of HMPAO was obtained from Nihon Medi-Physics Co., Ltd. (Tokyo, Japan). The animal experiments in this study were performed in accordance with institutional and national guidelines regarding animal care and approved by the Animal Care and Use Committee of the Hamamatsu University School of Medicine. Male SD rats (8 w) supplied by Japan SLC Co. (Hamamatsu, Japan) were housed under light/dark 12-h cycles and had free access to food and water.

### Preparation of ^99m^Tc-HMPAO

Approximately 1.11 GBq freshly eluted sodium [^99m^Tc]pertechnetate (5 mL) was added to the reaction vial of the HMPAO kit, and the reaction vial was gently swirled for a few seconds. Radiochemical purity was determined using a paper chromatography strip (Tec-Control Chromatography Strips for Bicisate and Exametazine; Biodex, Shirley, NY, USA) with ethyl acetate used as the mobile phase. Radioactivity in the fraction of the strips was determined with an auto-well gamma counter (1480 WIZARD^2^ 3, Perkin Elmer, Waltham, MA, USA). ^99m^Tc-HMPAO was injected into the rats within 30 min after its preparation. The radiochemical purity of ^99m^Tc-HMPAO just before injection was 90.5% ± 2.2%.

### Animal preparation

Rats (*n* = 13) were fasted for 15 h and then administered pentobarbital (50 mg/kg) intraperitoneally. The subsequent experiments were performed under pentobarbital anesthesia. A polyethylene catheter (i.d. 0.5 mm, o.d. 0.8 mm) was inserted into the femoral artery and filled with heparin saline solution (10 IU/mL) for blood sampling. Acetazolamide (0 mg/kg (*n* = 8), 25 mg/kg (*n* = 2), or 50 mg/kg (*n* = 3)) was then injected intravenously 15 min before ^99m^Tc-HMPAO administration.

### Experimental protocol

^99m^Tc-HMPAO (~ 185 MBq) was injected intravenously into the rats. Just after the ^99m^Tc-HMPAO injection, dynamic chest coronal planar scans were performed dorsally for 1 min using a small animal PET/SPECT/CT system (FLEX; Gamma Medica Ideas, Northridge, CA, USA) equipped with single pinhole collimators (1 mm). Arterial blood (approximately 100 μL per sample) was collected at 2, 6, 10, 14, 18, 22, 26, 30, 34, 38, 42, 46, 50, 54, and 58 s post-injection of ^99m^Tc-HMPAO. After a CT scan of the head, SPECT scan was performed using the small animal FLEX system equipped with multiple pinhole collimators (1 mm) from 15 to 57 min post-injection of ^99m^Tc-HMPAO, since radioactivity concentration in the brain is steady from 14 s to 60 min post-injection of ^99m^Tc-HMPAO [3]. SPECT data acquisition was performed at 30 s per projection with stepwise rotation of 64 projections over 360°. SPECT images were reconstructed using three-dimensional-ordered subset expectation maximization algorithms with 8 subsets and 10 iterations. After SPECT scanning, the rats were immediately sacrificed by decapitation, and the brain was removed and weighed. ^99m^Tc radioactivity concentrations in the arterial blood samples (C_Blood_-sampling) and brain (C_Brain_-sampling) were measured with the auto-well gamma counter.

Phantom studies were performed using a 14.1 mm diameter cylindrical phantom with known ^99m^Tc concentration (~ 11.1 MBq/mL) to mimic the size of rat heart and brain. Planar and SPECT imagings of the phantom were performed under the same conditions as already described rat heart and brain imagings. The region of interest (ROI) on planar images and the volume of interest (VOI) were manually drawn over the whole phantom to cross-calibrate between the radioactivity concentrations obtained from the planar or SPECT images, and auto-well gamma counter.

### CBF calculation using arterial blood and brain sampling (conventional procedure)

CBF was calculated using the microsphere method, as previously described [3], with slight modification. Briefly, C_Blood_-sampling at 14–60 s was fitted to a biexponential curve by the least-squares method to estimate the arterial blood radioactivity concentration at 19 s post-injection according to previous results [3]. C_Blood_-sampling was integrated using the trapezoidal approximation to obtain the area under the curve (AUC) until 19 s post-injection of ^99m^Tc-HMPAO (AUC_Blood_-sampling). C_Brain_-sampling quantified by the auto-well gamma counter was divided by the AUC_Blood_-sampling value to calculate the CBF using arterial blood and brain sampling (CBF-sampling).

### CBF calculation using planar and SPECT imaging

**Fig. 1:**
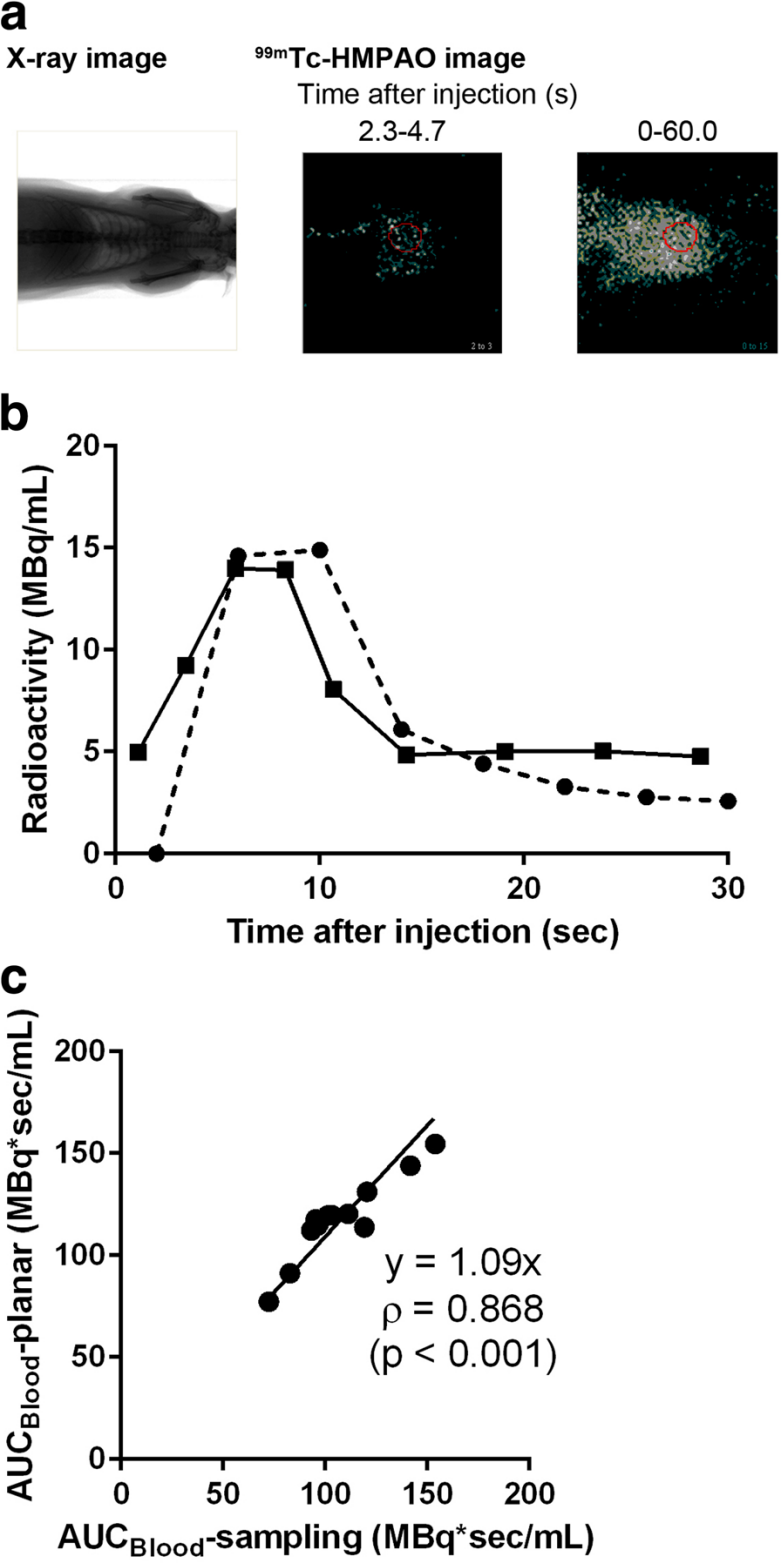
Input function of technetium-99m hexamethylpropyleneamine oxime (^99m^Tc-HMPAO). **a** Typical chest planar radiographic image (left panel) and ^99m^Tc-HMPAO images (middle and right panels) from the same rat. Region of interest (ROI) was manually surrounded on the ^99m^Tc-HMPAO-planar image by a red circle using the corresponding radiographic image as a reference. **b** Typical time–activity curves of the radioactivity concentration in arterial blood samples (C_Blood_-sampling, dashed line) and in the heart on planar images (C_Blood_-planar, solid line). **c** Comparison of integrated radioactivity concentrations in arterial blood samples (AUC_Blood_-sampling) and that in the heart on planar images (AUC_Blood_-planar) until 19 s post-injection of ^99m^Tc-HMPAO. Correlation between the AUC value determined by arterial blood sampling and that by chest dynamic planar imaging was assessed using Spearman’s rank correlation coefficient

**Fig. 2:**
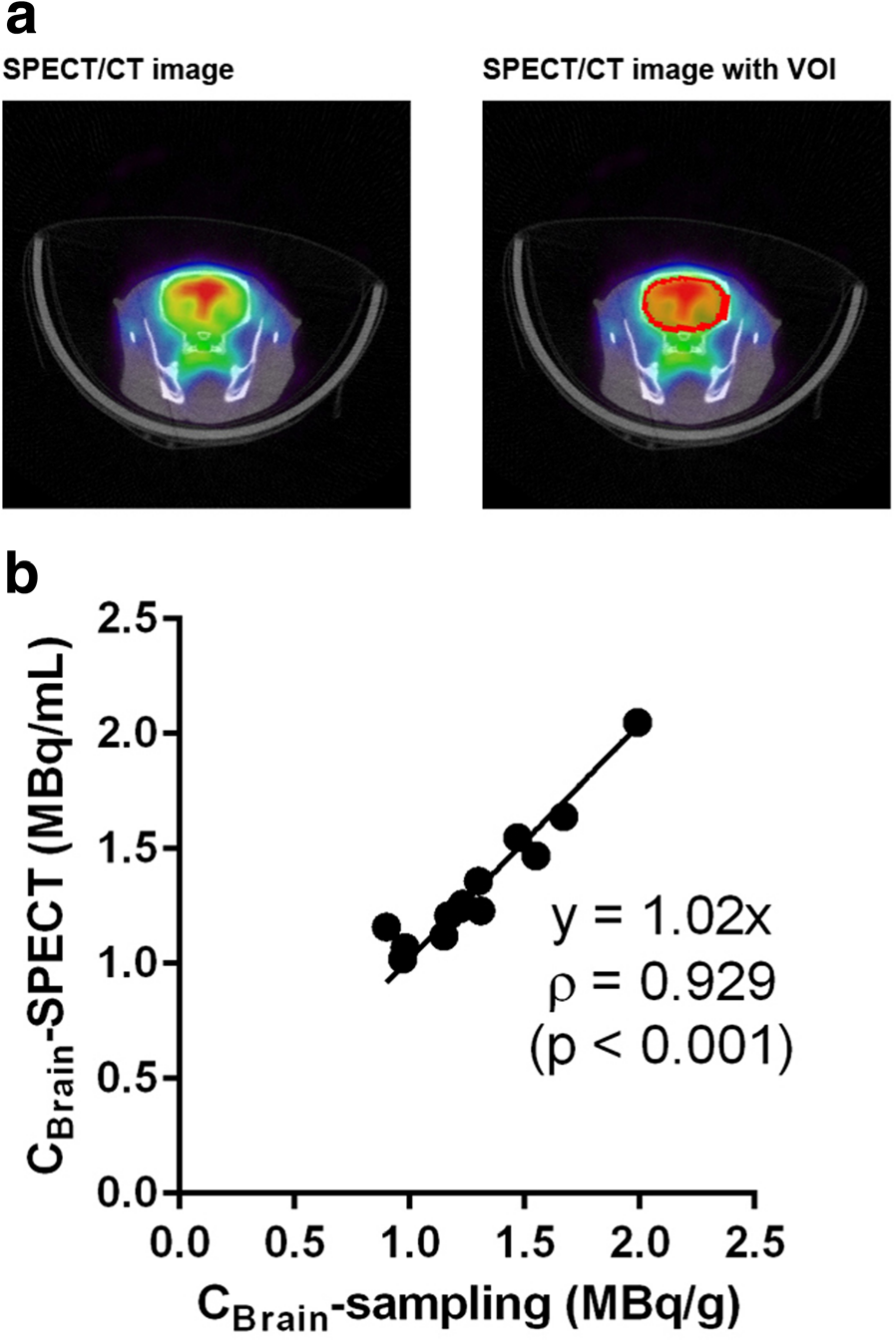
Radioactivity concentration in the brain (C_Brain_) after ^99m^Tc-HMPAO injection. **a** Typical single-photon emission computed tomography/computed tomography (SPECT/CT) fusion transversal image of rat brain during 15–57 min post-injection of ^99m^Tc-HMPAO (left panel) and the manually drawn volume of interest (VOI) (red circle, right panel). **b** Comparison of radioactivity concentration in brain determined by resection just after SPECT (C_Brain_-sampling) and that by SPECT 15–57 min post-injection of ^99m^Tc-HMPAO (C_Brain_-SPECT). Correlation between the concentration determined by resection and that by SPECT imaging was assessed using Spearman’s rank correlation coefficient

The ROI was manually drawn on the heart in the chest planar image using the radiographic planar image of the same rat as a reference (Fig. 1a), and the mean radioactivity concentration in the ROI was quantified (C_Blood_-planar). C_Blood_-planar was integrated using the trapezoidal approximation to obtain the AUC until 19 s post-injection of ^99m^Tc-HMPAO (AUC_Blood_-planar). Head CT and SPECT images were combined, and the VOI was manually drawn over the whole brain (Fig. 2a). C_Brain_ was quantified as the mean concentration in the VOI (C_Brain_-SPECT). C_Brain_-SPECT was divided by AUC_Blood_-planar to obtain CBF-imaging. CBF-imaging was also compared with the CBF calculated from C_Brain_-SPECT and AUC_Blood_-sampling to estimate the effects on CBF calculation between AUC_Blood_-planar and AUC_Blood_-sampling.

### Statistical analysis

The associations between AUC_Blood_-sampling and AUC_Blood_-planar, C_Brain_-sampling and C_Brain_-SPECT, and CBF-sampling and CBF-imaging were calculated using Spearman’s rank correlation coefficients.

## Results

### Input function measured from dynamic planar images vs. blood sampling

The time–activity curves for the heart measured from dynamic chest planar images and in arterial blood of the same rat measured from blood samples are shown in Fig. 1b. Both time–activity curves showed the highest concentration of ^99m^Tc at 5–10 s post-injection and followed time-dependent decreased radioactivity. AUC_Blood_-planar showed a good correlation with AUC_Blood_-sampling (Fig. 1c) (*n* = 13, *ρ* = 0.868, *p* < 0.001), and the regression line was nearly *y* = *x* (slope = 1.09).

### Brain radioactivity measured from SPECT images vs. resected samples

Figure 2b compares C_Brain_ obtained from SPECT images and resected brain samples. C_Brain_-SPECT was well correlated with C_Brain_-sampling (*n* = 13, *ρ* = 0.929, *p* < 0.001), and the regression line was nearly *y* = *x* (slope = 1.02).

### Noninvasively quantified CBF vs. conventionally quantified CBF

**Fig. 3:**
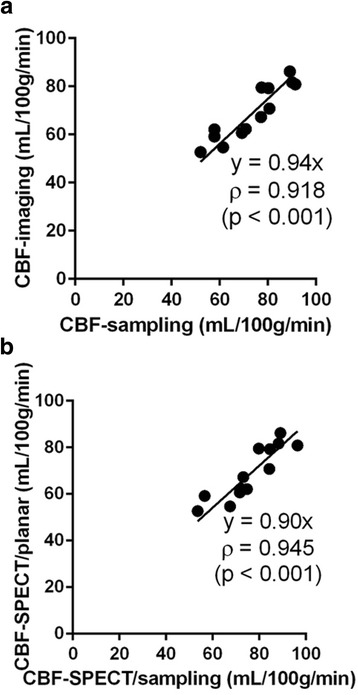
CBF quantification. **a** Comparison of conventionally quantitated CBF (CBF-sampling) calculated by dividing C_Brain_-sampling by AUC_Blood_-sampling and noninvasively quantitated CBF (CBF-imaging) calculated by dividing C_Brain_-SPECT by AUC_Blood_-planar. Correlation between CBF-sampling and CBF-imaging was assessed using Spearman’s rank correlation coefficient. **b** Comparison of CBF (CBF-SPECT/sampling) calculated by dividing C_Brain_-SPECT by AUC_Blood_-sampling and noninvasively quantitated CBF (CBF-SPECT/imaging) calculated by dividing C_Brain_-SPECT by AUC_Blood_-planar. Correlation between CBF-sampling and CBF-imaging was assessed using Spearman’s rank correlation coefficient

Figure 3a shows the correlation between CBF-imaging, which is noninvasively quantified using AUC_Blood_-planar and C_Brain_-SPECT, and CBF-sampling, which is conventionally quantified using AUC_Blood_-sampling and C_Brain_-sampling. CBF-imaging showed a good correlation with CBF-sampling. (*n* = 13, *ρ* = 0.918, *p* < 0.001). The regression line was nearly *y* = *x* (slope = 0.94).

### The effects on CBF quantification between dynamic planar images and blood sampling

Figure 3b shows the correlation between CBF-imaging and CBF quantified using AUC_Blood_-sampling and C_Brain_-SPECT (CBF-SPECT/sampling). CBF-imaging showed a good correlation with CBF-SPECT/sampling (*n* = 13, *ρ* = 0.945, *p* < 0.001). The regression line was nearly *y* = *x* (slope = 0.90).

## Discussion

Rat CBF could be quantified by a microsphere method in which C_Brain_ at 5 min post-injection is divided by the integrated arterial blood radioactivity concentration until 19 s post-injection of ^99m^Tc-HMPAO (AUC_Blood_) [3]. In the present study, AUC_Blood_ and C_Brain_ were noninvasively estimated using planar imaging and SPECT, respectively. Using the noninvasively estimated AUC_Blood_-planar and C_Brain_-SPECT, rat CBF was quantified (CBF-imaging) and compared with conventionally quantified rat CBF (CBF-sampling).

C_Blood_ was estimated using dynamic planar imaging of the heart-blood pool. To quantify the radioactivity concentration in arterial blood as an input function via chest dynamic planar images, the ROI should be drawn only on the left ventricle cavity. However, in this study, we drew the ROI over the whole heart to calculate the blood radioactivity concentration in the heart (C_Blood_-planar) as an input function, because rat left ventricle cavity is too small and too complicated to confirm by two-dimensional planar images. Hence, there could be some differences between C_Blood_-sampling and C_Blood_-planar values. In addition, planar imaging could underestimate the radioactivity concentration because of scatter and attenuation of gamma rays [12]. Despite these differences, C_Blood_-planar was identical to C_Blood_-sampling until 19 s post-injection of ^99m^Tc-HMPAO (Fig. 1b). AUC_Blood_-planar was well correlated with AUC_Blood_-sampling (Fig. 1c), suggesting that dynamic chest planar imaging could estimate the AUC_Blood_ until 19 s post-injection, which is required to quantify the rat CBF using ^99m^Tc-HMPAO [3]. These results indicate that the differences between arterial blood sampling and chest planar imaging might be negligible for estimating AUC_Blood_ until 19 s post-injection under the experimental conditions of the present study.

C_Brain_ was determined by SPECT. Because our previous study showed that C_Brain_ was quite steady from 14 s to 60 min post-injection of ^99m^Tc-HMPAO [3], SPECT would be well suited for quantitative evaluation of C_Brain_ within this period. Figure 2b shows that C_Brain_-SPECT at 15–57 min post-injection was identical to C_Brain_-sampling determined just after SPECT imaging. This result suggests that scatter and attenuation of gamma rays would be negligible for C_Brain_ quantitation by SPECT under these conditions, and SPECT could determine C_Brain_ after ^99m^Tc-HMPAO injection. Considering that C_Brain_ is steady from 14 s to 60 min post-injection of ^99m^Tc-HMPAO [3], C_Brain_-SPECT at 15–57 min post-injection of ^99m^Tc-HMPAO could be equal to C_Brain_ at 5 min post-injection, which is required for quantitation of rat CBF by the ^99m^Tc-HMPAO microsphere method [3].

Thus, rat CBF was calculated using a microsphere method in which C_Brain_ was divided by AUC_Blood_ until 19 s post-injection. CBF-imaging, which is obtained by dividing C_Brain_-SPECT by AUC_Blood_-planar, was identical to conventionally estimated CBF-sampling using C_Brain_-sampling and AUC_Blood_-sampling, suggesting that rat CBF could be quantitatively estimated using ^99m^Tc-HMPAO-planar and SPECT imaging without sampling arterial blood. Thus, noninvasive quantitation of rat CBF could enhance the repeatable CBF assessment without the physiological damage due to arterial cannulation and blood sampling.

## Conclusions

We propose a noninvasive procedure for quantitating rat CBF using ^99m^Tc-HMPAO. Rat CBF values, which were noninvasively quantified using chest dynamic planar imaging and head SPECT, were nearly identical to conventionally determined rat CBF using arterial blood and brain sampling. These findings indicate that rat CBF could be noninvasively and quantitatively assessed by combining ^99m^Tc-HMPAO-planar imaging and SPECT, thereby avoiding arterial blood sampling.
